# Combustion Enhancement of Gel Propellant Containing High Concentration Aluminum Particles Based on Carbon Synergistic Effect

**DOI:** 10.3390/gels10020089

**Published:** 2024-01-24

**Authors:** Jiyuan Chen, Hui Zhao, Weifeng Li, Haifeng Liu

**Affiliations:** National Energy Coal Gasification Technology Research and Development Center, Shanghai Engineering Research Center of Coal Gasification, East China University of Science and Technology, Shanghai 200237, China; 10182671@mail.ecust.edu.cn (J.C.); liweif@ecust.edu.cn (W.L.); hfliu@ecust.edu.cn (H.L.)

**Keywords:** metallized gel fuel, aluminum agglomeration, carbon synergistic effect, combustion enhancement, rheology

## Abstract

The addition of aluminum particles to gel propellants can improve combustion performance. However, the agglomeration of aluminum during the combustion process can result in a series of negative effects. In this paper, the aluminum agglomeration inhibition method of gel propellant based on carbon synergistic effect is proposed. Carbon particles exhibit excellent combustion properties, and the gaseous product CO_2_ generated during combustion can mitigate the agglomeration of aluminum. The research demonstrates that incorporating carbon particles into aluminum-containing gel effectively reduces the incomplete combustion of aluminum particles and increases the volumetric calorific value of the gel. When the mass fraction of carbon is 5 wt%, the volume calorific value of the gel reaches the highest. Meanwhile, the rheological experiments show that the addition of carbon particles can improve the shear-thinning properties of the gel, which is beneficial to the atomization and combustion processes of the gel.

## 1. Introduction

In recent years, gels have been increasingly applied in energy, aerospace, chemistry, material, medicine, etc. [1,2,3,4,5,6]. Gels are colloids in which the liquid medium has become viscous enough to behave more or less as a solid. When subjected to shear stress exceeding the yield stress, gels demonstrate shear thinning characteristics.

Gel propellant is a liquid propellant that incorporates a certain amount of gelling agents, such as silica [7,8], hydroxypropyl cellulose (HPC) [9], and Thixatrol-ST [10], so that it becomes a colloid mixture at normal temperature and pressure. Gel propellant not only inherits the characteristics of high specific impulse, multiple start-ups, and adjustable thrust of liquid propellant but also has the advantages of solid propellants, such as non-leakage, long-term storage, easy use, and maintenance [11]. Gel engine has broad development and application prospects in the field of missile weapons and space thrusters and has become a new propulsion technology widely studied [12,13,14,15].

Gel propellants exhibit different flow and deformation characteristics under external pressure or shearing. The rheological properties of gel propellants suitable for rocket engines should be satisfied: in the storage and movement process, it appears as a solid with a large enough yield stress to prevent flow and deformation leakage, while in the working process, it appears as a fluid, the apparent viscosity should decrease with the increase of the shear rate. Therefore, gel propellants are the typical non-Newtonian pseudoplastic fluids whose rheological properties can usually be characterized by Bingham, Power–Law (PL), Herschel–Bulkley (HB), and Carreau, etc. [9,14,16,17,18,19].

In order to enhance the energy and density of gel propellant, energetic powdery particles, such as aluminum, magnesium, boron, and carbon, are usually mixed. The combustion characteristic of gels containing particles will also be significantly affected [20,21]. Among these particles, aluminum particles are extensively employed in propellants. However, research has revealed that gels containing aluminum particles does not burn completely, and the increase in aluminum content leads to a decrease in combustion efficiency [22]. Agglomeration of aluminum particles results in the formation of large agglomerates that are tens or even hundreds of times larger than the aluminum particles. Furthermore, the combustion product of aluminum particles can be divided into two components: one is the large-size alumina formed by the combustion of agglomerated aluminum, which is called Agglomerates. The remaining part is the small particle of alumina formed by aluminum vapor during combustion, which is called SOPs (Smoke oxide particles) [23]. In order to improve the combustion characteristics of aluminum in aluminum-containing propellants, optimizing the formulation of the propellant is an effective approach. Jiang [24] added 20 wt% graphene oxide (GO) to the micron-sized aluminum powder and coated it on the surface of aluminum particles for combustion experiments. The research found that the heat generated by GO combustion, the product rGO, and gaseous products (H_2_O, CO_2_) all contribute to promoting the combustion of aluminum powder. Liu [25] added graphene to the propellant, and studies have demonstrated that graphene can reduce aluminum agglomeration. As the content of graphene increases, the average size of large particles in the condensed combustion products decreases. By adding 0.5 wt% graphene, large agglomerates are almost eliminated. Certain fluorides have a remarkable effect on inhibiting the agglomeration of aluminum particles. Ao [26] investigated the impact of adding a new functionalized Fluorine-containing organic substance (FCOS) to aluminized solid propellants. With the addition of FCOS, the combustion rate of the propellant increases and produces more gas-phase combustion products, thereby reducing the size of aluminum aggregates. The energy performance of propellants has also been improved. In addition, some studies have explored using alloy powders to replace aluminum powder in propellants, such as Al-Mg alloy particles [27] and Al–Li alloys [28]. Therefore, the propellant formulation has a significant impact on the combustion properties of aluminum-containing propellants.

Currently, the focus of research lies in reducing the incomplete combustion of aluminum powder in aluminum-containing solid propellants. However, there has been little discussion about how to improve the incomplete combustion of aluminum powder in gel propellants. It remains to be studied. Therefore, after referring to the method of improving the incomplete combustion of aluminum powder in solid propellant, our research is devoted to improving the incomplete combustion of aluminum particles within gels.

Our research is focused on developing gel propellants with better energy performance and rheology, which can be used in the field of aerospace propellants. Previous studies have increased the addition of aluminum powder in ethanolamine gel to 50 wt%. However, the commonly used micron-sized aluminum powder has a serious agglomeration phenomenon; the excessive content of aluminum powder leads to a reduction of the combustion heat growth rate and significant incomplete combustion within the ethanolamine gel. Our research found that adding a small amount of carbon particles into the ethanolamine gel containing 50% Al powder can enhance the calorific value of the aluminum-containing gel and improve the combustion characteristics. Ethanolamine fuel, carbon particles, and aluminum powder were selected to prepare gel fuel. To investigate the impact of carbon particles on reducing the incomplete combustion of Al-containing gel, we conducted measurements on the heat of combustion of the gel and observed its combustion products using a scanning electron microscope. Additionally, we studied the rheological properties of the gel.

## 2. Results and Discussion

### 2.1. Combustion Heat Detection

**Figure 1:**
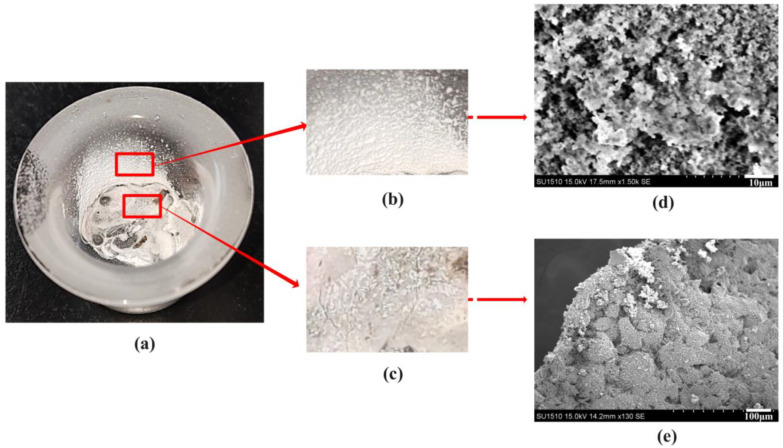
Combustion products of ethanolamine gel fuel and its drawing of partial enlargement. (**a**) Combustion products in a quartz crucible. (**b**) SOPs. (**c**) Agglomerates. (**d**) Microscopic topography of SOPs. (**e**) Microscopic topography of agglomerates.

The heat of combustion of the gel was measured using a 5E-AC8018 calorimeter. After the measurement, the quartz crucible was removed, and the large-size alumina formed by the combustion of agglomerated aluminum (agglomerates) and the small particle alumina formed by aluminum vapor during combustion (SOPs) could be seen, as depicted in Figure 1. The microscopic morphology of both agglomerates and SOPs can be seen by scanning electron microscopy. SOPs are white and fluffy, resulting from the stacking of small particles of alumina. The microscopic profile of agglomerates varies with the gel formulation and will be analyzed in Section 2.2.

According to the data from reference [29], the mass heat of the combustion of aluminum is 32 MJ/kg, and the volume calorific value is 85 MJ/kg. Ethanolamine, identified as a novel propellant [21], has a mass heat of combustion of 25.18 MJ/kg. As an organic gelling agent, the mass heat of combustion of agarose is 4.97 MJ/kg. Based on the density and combustion heat of each component within the gel, it can be calculated that the theoretical combustion heat of the gel is 27.77 MJ/kg.

The mass heat of combustion and the volume calorific value of the gel under different formulations are presented in Table 1. During the experiment, 0.3 g of gel is combusted in an oxygen bomb with a volume of 300 cm^3^ and a pressure of 3.5 MPa. The oxygen content in the combustion environment surpasses the requirements for complete fuel combustion, thereby eliminating the possibility of incomplete combustion resulting from inadequate oxygen supply. Due to incomplete combustion of aluminum, the actual measured results are smaller than theoretical values.

**Figure 2:**
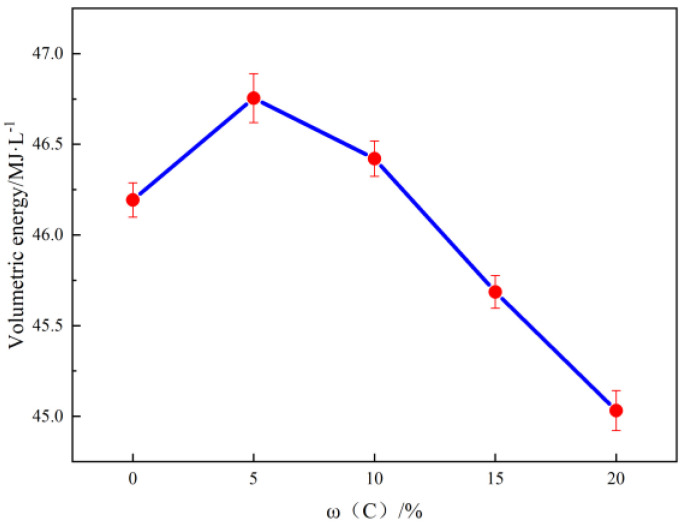
Relationship between carbon particle mass fraction and volume calorific value of gel fuel. Bars donate S.D.

It is evident that an increase in carbon content leads to a corresponding increase in the mass heat of combustion of the gel fuel. After calculating the volume calorific value of the gel fuel through the density of the sample, it can be seen in Figure 2 that the volume calorific value of the gel fuel first increases and then decreases. The volume calorific value of 50Al gel is measured at 46.193 MJ·L^−1^, and after mixing with 5% carbon particles, it reaches 46.755 MJ·L^−1^, which is 1.21% higher than 50Al gel. When the amount of carbon particles added reaches 10%, the volume calorific value remains higher than that of 50Al gel at 46.421 MJ·L^−1^, indicating that mixing an appropriate amount of carbon particles can enhance the volume calorific value of the gel. The phenomenon can be attributed to two factors: Firstly, the carbon particles themselves possess a relatively high calorific value (33 MJ/kg), thereby enhancing the overall combustion heat of the gel fuel. Secondly, the aluminum powder utilized in this study exhibits severe agglomeration, resulting in incomplete combustion of the aluminum-containing gel during combustion, and the addition of carbon particles can mitigate this issue. The specific principles will be discussed in Section 2.3. With the addition of more carbon particles, the overall density of the gel system decreases, consequently leading to a reduction in the volume calorific value of the gel fuel. This phenomenon can be attributed to the low density exhibited by carbon particles; an excessive amount of these particles diminishes the gel’s density and subsequently lowers its mass heat of combustion. Therefore, the quantity of carbon particles introduced must be carefully controlled; otherwise, it will reduce the combustion performance of the gel fuel.

### 2.2. Scanning Electron Microscopy Analysis of Combustion Products

**Figure 3:**
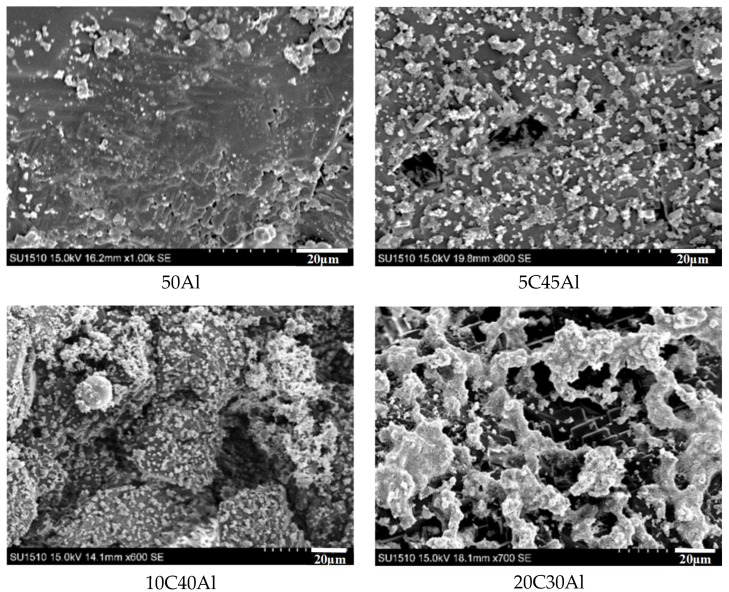
Scanning electron microscope images of combustion products of gel fuel.

The scanning electron microscope image in Figure 3 clearly reveals the solidified black-gray alumina aggregates resulting from burning, as well as the fluffy white small particle alumina formed through the combustion of aluminum vapor on the surface (SOPs). When the mass fraction of aluminum powder is 50 wt%, the alumina agglomerates formed after combustion exhibit a relatively smooth surface, and the surface is covered with a small number of white alumina particles (SOPs). Compared to the gel containing 50 wt% aluminum powder, the surface of alumina agglomerates formed by gel combustion is rougher and has a small number of pores upon incorporation of 5% carbon particles. This phenomenon can be attributed to the CO_2_ gas generated by the mixed carbon particles after combustion, which causes damage to alumina aggregates. There are also more SOPs on the surface, indicating that the production of carbon dioxide promotes the flow of aluminum vapor during combustion, resulting in more SOPs. When the mixed carbon particles content reaches 10 wt%, the surface of alumina aggregates formed by gel combustion exhibits increased roughness, and the number of pores and SOPs also increased. Upon addition of 20 wt% carbon particles, the surface structure is more complex, a large number of pores are distributed on the surface, and more SOPs with larger volumes can be observed.

**Figure 4:**
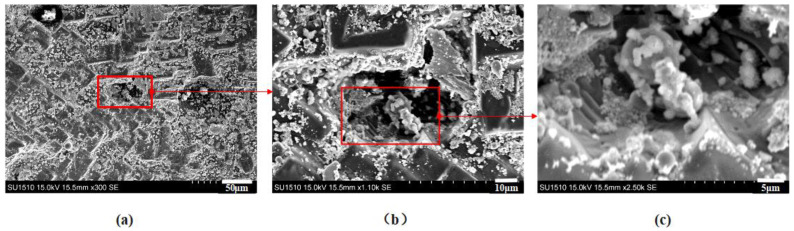
Scanning electron microscope image of holes (5C45Al). (**a**) Combustion products of gel fuel (5C45Al). (**b**) The hole image magnified by a factor of 1100. (**c**) The hole image magnified by a factor of 2500.

Through electron microscopy in Figure 4, it can be observed that the combustion products of carbon-containing gel fuel exhibit the presence of multiple holes, which are uncommon in 50Al gel. The carbon particles in the gel burn and produce CO_2_, which expands and breaks down the alumina agglomerates, resulting in these holes. These pores facilitate the release of unreacted aluminum, thereby enabling continuous combustion reactions. The presence of SOPs distributed within these pores indicates the complete combustion of aluminum particles.

The aforementioned results demonstrate that as the quantity of carbon particles increases, the CO_2_ gas can effectively destroy the interior of alumina aggregates, diminish the agglomeration of aluminum particles while generating a multitude of holes, and enhance the combustion efficiency of aluminum powder.

### 2.3. Mechanism of Carbon Particles Improving Aluminum Agglomeration

**Figure 5:**
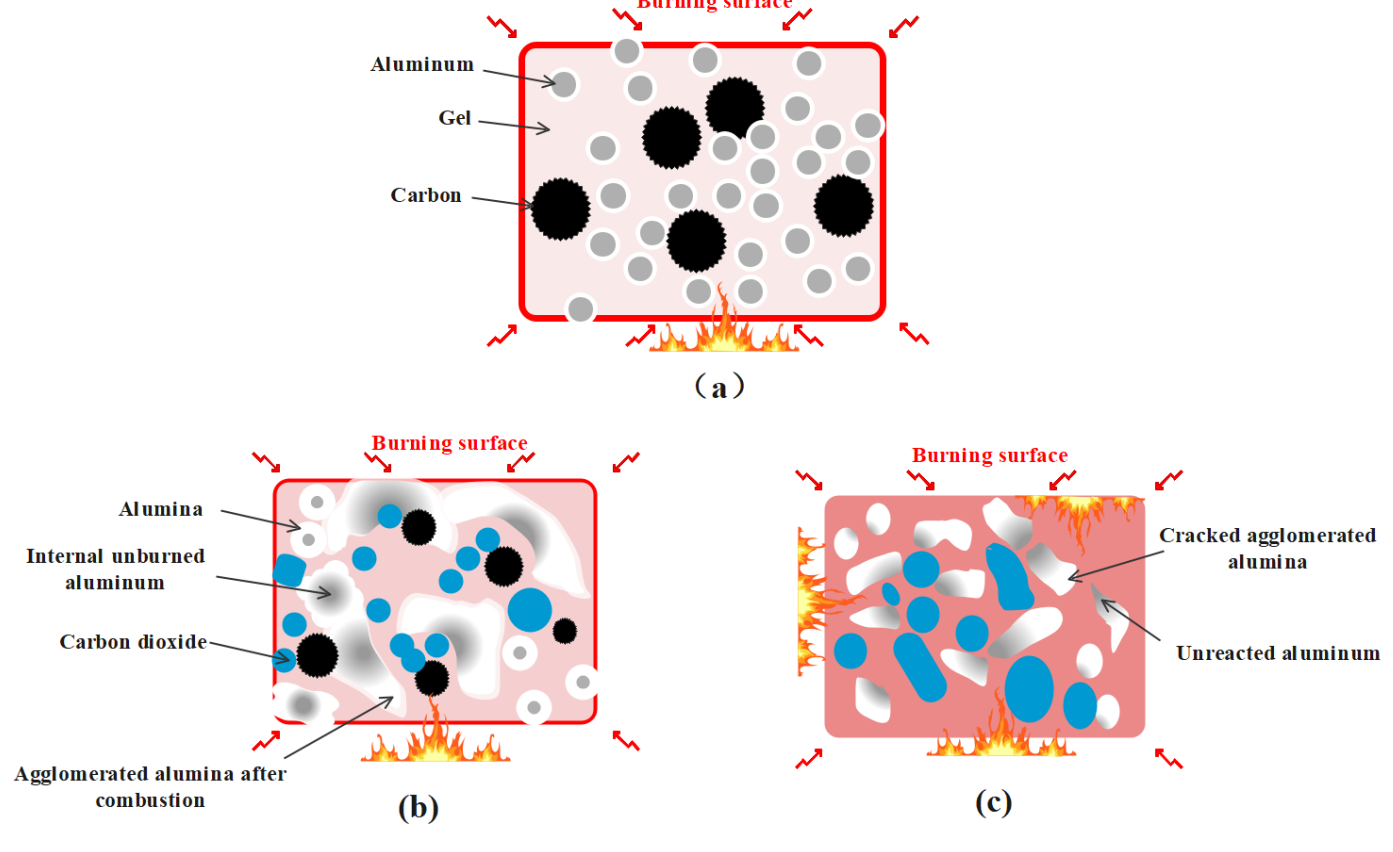
Mechanism diagram of carbon particles reducing incomplete combustion of aluminum particles. (**a**) The gel is ignited and forms a burning surface. (**b**) Melting, agglomeration, and oxidation of aluminum particles at high temperatures. (**c**) Large agglomerates of aluminum are destroyed by carbon dioxide.

The schematic diagram in Figure 5 illustrates the combustion process of a gel containing carbon particles and aluminum particles in an oxygen environment. Carbon particles and aluminum particles are distributed inside the gel. After ignition, the surface of the gel undergoes combustion, gradually propagating towards its interior, resulting in a rapid increase in temperature within a short period (Figure 5a). As the combustion continues, the aluminum particles gradually melt and gather together with the temperature rising. With the rupture of the outer layer of the gel, the internal particles come into contact with oxygen and undergo combustion. Alumina is formed by the combustion of aluminum particles and covered on the surface of aluminum. The aluminum coalesces into sizable alumina aggregates following combustion, impeding the further combustion of internal aluminum. At the same time, carbon particles also burn and produce carbon dioxide (Figure 5b). As combustion progresses, these carbon particles undergo a complete reaction, generating a significant amount of carbon dioxide that rapidly expands at high temperatures and disrupts alumina aggregates. This effectively retards aluminum agglomeration during combustion. At the same time, the unreacted aluminum is released (Figure 5c), which comes into contact with the oxygen in the surrounding environment and undergoes combustion.

### 2.4. Rheological Study

#### 2.4.1. Apparent Viscosity Test

As a shear-thinning non-Newtonian fluid, the apparent viscosity of gel decreases with the increase of shear rate. The investigation into the shear thinning characteristics of gel propellant has significant reference value for its storage, flow, and atomization. Jyoti [30] investigated the shear-thinning behavior of ethanolamine-based gel propellant across shear rates ranging from 1 to 1000 s^−1^. The experimental results show that the viscosity of the gel increases gradually at a very low shear rate and does not begin to flow until it reaches the yield stress. In this case, the viscosity is higher than 100 Pa·s. Subsequently, the gel exhibits the characteristic of shear thinning as the shear rate increases. In addition, Jyoti [31] discovered that when aluminum particles with a mass fraction of 20 wt% were added to the ethanol gel, the viscosity of the system increased from approximately 100 Pa·s to over 1000 Pa·s. Consequently, compared to pure gel systems, the addition of metal particles can greatly improve the viscosity.

**Figure 6:**
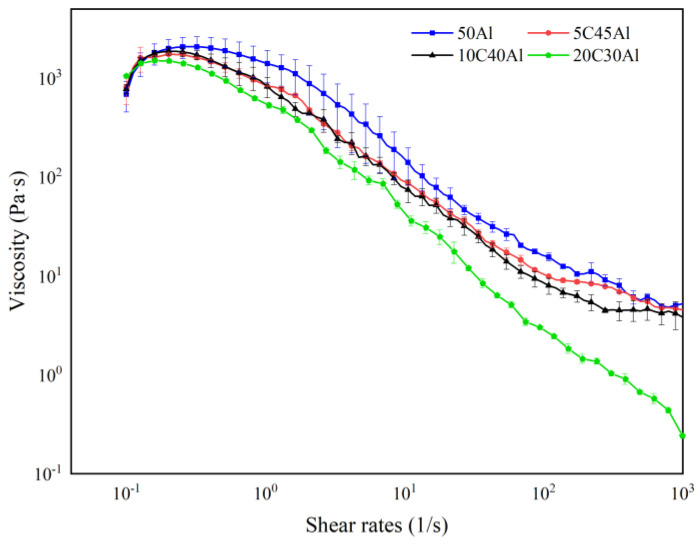
Relationship between gel viscosity and shear rate. Bars donate S.D.

The relationship between viscosity and shear rate of gels containing different energetic particles is illustrated in Figure 6. It can be observed from Figure that as the shear rate increases, the gels in all four formulations will first shear and thicken to the yield point, followed by a gradual decrease with a further increase in shear rate. These are consistent with references [30,31]. The difference in viscosity is primarily attributed to the type of gelling agent and the mass fraction of energetic particles within the gel formulation. When subjected to lower shear rates, substituting carbon particles for aluminum particles has minimal impact on the viscosity of the gel, and the viscosity of three gels containing carbon powders is approximately equivalent to that containing 50 wt% aluminum.

According to the characteristics of the viscosity curve, the Power-Law fluid constitutive equation is further employed to depict the correlation between the viscosity of the gel fuel and the shear rate in the range of medium shear rate (1–10^3^ s^−1^). The Power–Law equation [32] can be expressed as follows:

*η* = *Kγ ^n^*^−1^ (1)

where *η* is the viscosity of the gel, Pa·s; *K* is the flow consistency index, and Pa·s*^n^*; *n* is the power–law exponent. The fitting results are shown in Table 2.

The size of the viscosity coefficient *K* reflects the level of viscosity in the gel system. As the carbon content increases, *K* gradually decreases, indicating that the viscosity of the system decreases, which aligns with the conclusion drawn from the viscosity curve. In Newtonian fluid, the Power-Law index *n* is equal to 1. Non-Newtonian fluid with *n* < 1 is called pseudoplastic fluid, which exhibits shear thinning behavior. And the more *n* deviates from 1, the more significant the shear thinning characteristic of pseudoplastic fluid is. It can be observed from Table 2 that the addition of carbon particles enhances the shear-thinning properties of the gel. At high shear rates, an increase in the amount of carbon particles leads to a decrease in viscosity for the gel system. When more carbon particles (20%) replace aluminum particles, the viscosity of the gel system will decrease significantly. This is attributed to the lower density and loose particle microstructure of carbon particles compared to aluminum powder, resulting in weaker bonding between particles and gels. As the content of carbon particles increases relative to pure aluminum gel, internal resistance within the gel decreases, leading to a reduced viscosity.

#### 2.4.2. Thixotropy

**Figure 7:**
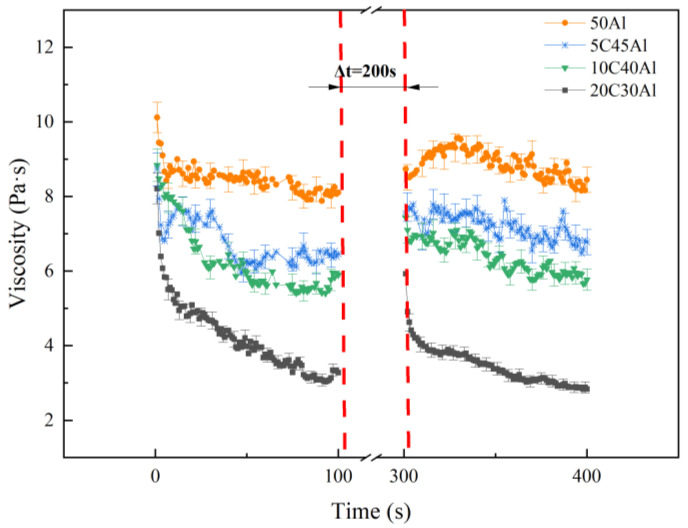
Thixotropy curves of gels with different formulations.

When the gel system is subjected to the shear force, the internal network of the gel is also sheared, and the gel system will be destroyed under the shear force. However, upon removal of the shear force, the internal network can regenerate and reform the gel system through thixotropy. In this study, the 3ITT (three Interval Thixotropic Test) was used to evaluate the thixotropy of ethanolamine gel. The gel was exposed to a constant shear rate of 300 s^−1^ during 0–100 s. Then, the shear rate between 100–300 s was removed, and the gel was allowed to recover by itself for 200 s. Finally, a constant shear rate of 300 s^−1^ was applied again from 300–400 s. The change curve of the viscosity of the gel with time is shown in Figure 7.

It can be observed from Figure 7 that, under a constant shear rate of 300 s^−1^, the viscosity of the gel in all four formulations first decreased rapidly and then gradually stabilized. After undergoing self-recovery for 200 s, the four gels displayed varying degrees of recovery. When the shear stress was applied again, the viscosity exhibited a similar trend as observed during the first 100 s, but none of them could return to their initial states. Based on the recovery ratio of the gel in Table 3, it can be concluded that the recovery ratio of the gel increases with the carbon content. Compared to aluminum powder, carbon particles exhibit a lower density and looser particle microstructure, and the bonding between particles and gel is weaker than aluminum, so the gel mixed with carbon particles demonstrates enhanced recovery capabilities. Since the recovery of the gel structure will increase the viscosity and energy loss of the gel fuel during practical application, the lower thixotropy levels are beneficial to the subsequent shear thinning and atomization process of the gel fuel. The excessive addition of carbon particles in practical applications is therefore deemed inappropriate.

#### 2.4.3. Amplitude Sweep

**Figure 8:**
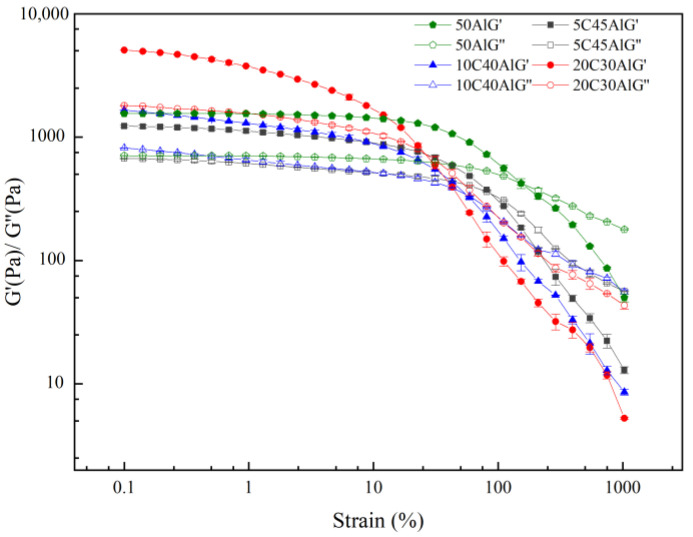
Amplitude scan curves of different gel fuel samples.

The result of the amplitude sweep is illustrated in Figure 8. With the increase of gel strain, the elastic modulus G’ initially stabilizes and subsequently exhibits a gradual decline. The range where G’ and G” vary within 5% is called the linear viscoelastic region (LVR) of the gel fuel, and the point where the G’ of the gel decreases is called the critical strain. As the critical strain increases, so does the extent of the linear viscoelastic region (LVR).

The critical strain of gels with different formulations is presented in Table 4. As the amount of carbon particles increases, there is a gradual decrease in the critical strain of gel fuel, accompanied by a reduction in the corresponding linear viscoelastic region. Upon reaching the critical strain, the molecular chains within the gel undergo elongation to their maximum extent along the flow direction, resulting in structural breakdown. Aluminum particles can form a strong binding force with the gel, leading to a higher critical strain. However, due to the loose microstructure of the carbon particles, the bonding between the particles and the gel is weak; with the addition of carbon particles and the reduction of aluminum particles, the bonding force between particles and molecular chains is reduced, making the gel more prone to relative slippage. The point at which the intersection of G’ and G” occurs is commonly referred to as the yield point. It can be observed from Figure 8 that the G’ of all four gels is higher than the G” before the yield point, indicating their solid characteristics during this stage. Beyond the yield point, the G” of the gel is higher than the G’, and the gel transitions to fluid properties. The yield point strain of gel fuel can be found in Table 4. The yield point strains in all samples exceed the critical strain, indicating that the internal grid structure of the four gels is disrupted during flow. The incorporation of carbon particles reduces the strain at the gel’s yield point, suggesting that compared to aluminum particles, carbon particles diminish the stability of gel fuel and render it more susceptible to structural failure under external forces. Moreover, when subjected to strong external forces, the shear-thinning characteristics become more pronounced.

#### 2.4.4. Frequency Sweep

**Figure 9:**
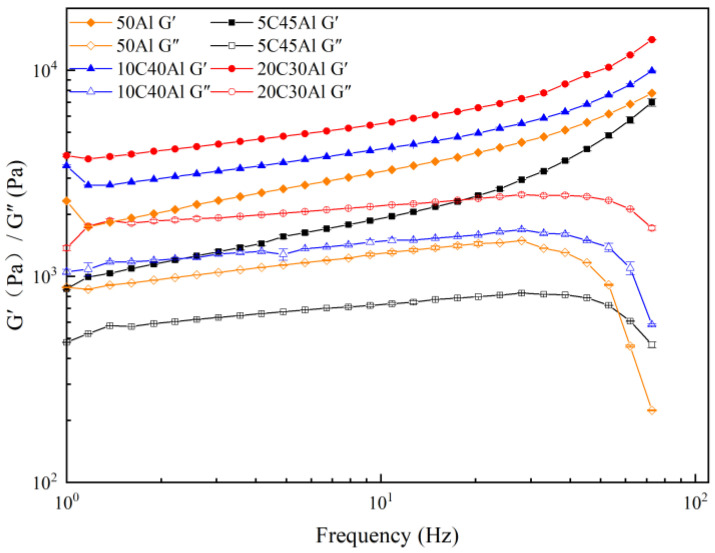
Relationship of storage modulus and loss modulus with frequency for different gel fuels.

The frequency sweep can be utilized to acquire the correlation between the viscoelasticity of the gel and the time scale. Higher frequencies correspond to strains on the sample with shorter time scales, while lower frequencies represent long timescale strains on the sample. The relationship between storage modulus and loss modulus of four gels was investigated through a frequency sweep analysis. Figure 9 illustrates the relationship between G’/G” and the frequency of four kinds of gels at 298 k and 1% strain. Within the frequency range of 1–100 Hz, the storage modulus of all four samples is higher than the loss modulus, indicating that the gel samples exhibit solid characteristics at lower strains. Furthermore, it is evident that both storage modulus and loss modulus values for 5C45Al gel are lower than those for pure 50Al gel, suggesting that incorporating a small amount of carbon particles reduces the mechanical strength of the gel. When more carbon particles are added, the storage modulus and loss modulus of 10C40Al and 20C30Al exhibit an increase compared to 50Al, and this increase is further enhanced with the addition of more carbon particles. It can be observed that the mechanical strength of the gel has been improved by mixing more carbon particles. Furthermore, as the oscillation frequency increases, all four samples demonstrate an elevation in their storage modulus. This trend suggests that at higher frequencies, the gel samples will exhibit pronounced elasticity, which will significantly impact subsequent gel atomization processes.

Through the rheological test, it can be found that adding a small amount of carbon particles (about 5%) can slightly improve the rheological properties of the gel propellant, making it more conducive to the subsequent atomization and other processes. However, an increase in the proportion of blended carbon particles leads to adverse effects. Although the gel with a carbon content of 20% exhibits improved shear thinning properties, it displays high thixotropy, poor stability, and excessive mechanical strength, which will affect subsequent atomization.

## 3. Conclusions

In this study, we investigated the impact of carbon powder on the combustion and rheological properties of aluminum-containing gel fuels. Scanning electron microscope images reveal that The CO_2_ gas produced by the combustion of carbon powder forms a large number of fluffy pores in the agglomerates, which makes the large-size aggregates of aluminum break so that the active aluminum wrapped by the aggregates can react. The agglomeration during aluminum combustion is mitigated. And the energy performance of gel fuel is improved, which has been confirmed in the combustion heat test.

The rheological experiments demonstrate that the addition of a small quantity of carbon particles results in a slight reduction in the viscosity of the gel system. Moreover, it diminishes the linear viscoelastic region of the gel, thereby compromising its stability and augmenting its shear-thinning properties. This makes the gel system easier to atomize. However, the excessive addition of carbon particles will result in increased thixotropy and mechanical strength of the system, thereby posing challenges to the subsequent gel atomization process.

Thus, incorporating a small amount of carbon particles (approximately 5 wt%) into the aluminized gel fuel can enhance the combustion performance of the propellant, mitigate the agglomeration effect of aluminum particles, and the rheological property plays a positive role in the subsequent atomization process of gel fuel.

## 4. Material and Methods

### 4.1. Materials

Ethanolamine fuel with a density of 1.013 g/cm^3^ (C2H7NO, Analytical Pure) was purchased from Shanghai McLean Biochemical Technology Corporation, Shanghai, China. Carbon particles, with a density of 1.800 g/cm^3^, were purchased from Shanghai New Platinum Chemical Technology Corporation, Shanghai, China. Agarose (Analytical Pure) was purchased from Qingdao Tenglong Microwave Technology Corporation, Qingdao, China. Aluminum particles, with a density of 2.700 g/cm^3^ and a characteristic particle size *D*_32_ (the ratio between the total volume of all particles and the total surface area) of 1 μm (analytically pure) were purchased from Angang Industrial Fine Aluminum Particle Corporation, Anshan, China.

### 4.2. Equipment and Instruments

The calorimeter 5E-AC8018 was provided by Changsha Kaiyuan Instrument, Changsha, China. The precision electronic balance ME303E was provided by Mettler Toledo, Zurich, Switzerland. The scanning electron micromirrors, SU1510 and S3400-N, were provided by Hitachi-Hightech (Shanghai) International Corporation, Shanghai, China. The oscillating agitator was provided by Shanghai Yuezhong Instrument, Shanghai, China. The rotary rheometer MCR 302 was provided by the Anton Paar (Shanghai) Trading Corporation, Shanghai, China.

### 4.3. Preparation of Gel

Firstly, 46.00 g of ethanolamine was added to the beaker, and 4.00 g agarose was weighed. Agarose was gradually dispersed into ethanolamine using a sieve. After that, evenly mixed aluminum/carbon particles (50 g in total) were added, and the sample was placed in an oscillating agitator for high-speed stirring (2000 r/min) for 30 min, then removed and sealed for 6 h to form a gel. The composition of each sample is presented in Table 5 (The sample number represents the mass ratio of carbon particles and aluminum particles in the proportion of 50 wt% particles of gel fuel).

### 4.4. Measurement and Characterization

#### 4.4.1. Combustion Heat Detection

The combustion heat of gel fuel was measured by an oxygen bomb calorimeter. The experimental procedure can be found in reference [33,34]. The volume of the oxygen bomb is 300 cm^3^. The 0.3 g sample was weighed in a quartz crucible, and the combustion heat of the sample was measured in an oxygen bomb tank at a pressure of 3.5 MPa. The experimental results were taken as the average value of three measurements.

#### 4.4.2. Scanning Electron Microscope (SEM)

A proper amount of gel samples burned in a calorimeter were frozen and dried in a freeze-drying machine. The treated samples were vacuum-gilded using a vacuum pump and subsequently transferred into the scanning electron microscope cavity. The microstructure and morphology of the gel combustion products were subsequently examined using a scanning electron microscope.

#### 4.4.3. Rheological Measurements

The rheological properties of the gel were measured by Anton Paar rheometer (MCR 302). The paddle rotor with a diameter of 22 mm is used for all measurements in this study. Before the test, gel fuel was added to the cylinder (test chamber), followed by the insertion of the paddle rotor. Then, the rheometer was operated to conduct the rheological test. The measurement result is taken as the average value of three times.

Rheological testing includes:

(1) Shear viscosity test: The rheometer operates in rotation mode. Control the shear rate of the rotor in the range of 0.1–1000 s^−1^. By gradually increasing the shear rate of the paddle rotor according to a logarithmic law starting from 0.1 s^−1^, we can accurately record and analyze the relationship between the shear rate and viscosity of gels;
(2) Thixotropic test: 3ITT (three Interval Thixotropic Test) [35] was used to detect the thixotropy of ethanolamine gel. A constant shear rate (300 s^−1^) was applied to the gel during 0–100 s. Remove the shear rate within 100–300 s and let the gel recover itself for 200 s. A constant shear rate (300 s^−1^) is still applied for 300–400 s. Finally, the curve of gel time and viscosity change is obtained;
(3) Amplitude sweeping test: Set the oscillation frequency of the rotor to 1 Hz, the strain range to 0.1–1000%, and record the storage modulus (G’) and loss modulus (G”).
(4) Frequency sweep test: constant strain is 1%, frequency is 1–100 Hz. Record the change of storage modulus (G’) and loss modulus (G”) at different frequencies.

## Figures and Tables

**Table 1:** The mass heat of combustion and volume calorific value of the gel fuel.

Sample	Mass Heat of Combustion/MJ·kg^−1^	Volume Calorific Value/MJ·L^−1^	Density g/cm^3^
50Al	25.578	46.193	1.805
5C45Al	26.423	46.754	1.769
10C40Al	26.787	46.421	1.732
15C35Al	26.930	45.686	1.696
20C30Al	27.128	45.031	1.659

**Table 2:** Power–Law fluid constitutive equation fitting table for gel samples.

Sample	*K*	*n*	R^2^
50Al	847.682	0.3602	0.99
5C45Al	738.579	0.3551	0.99
10C40Al	655.582	0.2729	0.99
20C30Al	516.438	0.1953	0.99

**Table 3:** Viscosity parameters of each sample before and after recovery.

Sample	Viscosity before Recovery/Pa·s	Viscosity after Recovery/Pa·s	Recovery Ratio/%
50Al	8.248	8.517	3.26
5C45Al	6.461	7.532	16.57
10C40Al	5.897	7.435	26.07
20C30Al	3.276	5.928	80.95

**Table 4:** The critical strain and yield point strain of the gel.

Sample	Critical Strain/%	Yield Point Strain/%
50Al	22.80	163.00
5C45Al	16.60	96.00
10C40Al	4.67	51.25
20C30Al	0.50	27.05

**Table 5:** Composition of each sample.

Sample	w/%			
	C_2_H_7_NO	Agarose	Al	C
50Al	46	4	50	0
5C-45Al	46	4	45	5
10C-40Al	46	4	40	10
15C-35Al	46	4	35	15
20C-30Al	46	4	30	20

## Data Availability

All data and materials are available on request from the corresponding author. The data are not publicly available due to ongoing research using a part of the data.
